# Synthesis, Structural Characterization, and Investigation of Biological Effects of Novel Benzothiazole‐Triazole Derivatives Designed as AKT Kinase Inhibitors

**DOI:** 10.1111/cbdd.70404

**Published:** 2026-09-17

**Authors:** Bilge Çiftçi, Zeynep Gülenç, Büşra Korkut Çelikateş, Derya Osmaniye

**Affiliations:** ^1^ Institute of Graduate Education Anadolu University Eskişehir Turkey; ^2^ Department of Medical Services Techniques Services, Vocational School of Health Services Bilecik Seyh Edebali University Bilecik Turkey; ^3^ Department of Pharmaceutical Toxicology, Faculty of Pharmacy Anadolu University Eskişehir Turkey; ^4^ Department of Pharmaceutical Chemistry, Faculty of Pharmacy Anadolu University Eskişehir Turkey; ^5^ Central Research Laboratory, Faculty of Pharmacy Anadolu University Eskişehir Turkey

**Keywords:** AKT, apoptosis, benzothiazole, breast cancer, PI3K

## Abstract

Breast cancer (BC) is one of the most common cancers in women worldwide, and the need for new, more effective treatment options remains. The PI3K/Akt signaling pathway and the aromatase enzyme are among the molecular targets that play a critical role in the development and progression of BC. In this study, novel benzothiazole‐triazole derivatives were synthesized, and their structures were confirmed using NMR and HRMS analyses. The cytotoxic activities of the eight synthesized compounds in the MCF‐7 bc cell line were evaluated using the MTT method, with NIH/3T3 cells used as a healthy cell model. The IC_50_ values of the compounds ranged from 10.186 to 52.020 μM, with compounds **5b**, **5f**, and **5h** showing the highest activity. Selectivity index results revealed that compound **5h** (SI = 5.986) exhibited the optimal selectivity profile along with strong anticancer activity. In enzyme inhibition studies, compound **5h** showed a strong inhibitory effect on Akt1, exhibiting activity close to the reference inhibitor with an IC_50_ value of 0.209 ± 0.011 μM. However, the lack of significant inhibition of aromatase enzymes indicated that the compound displayed a more selective inhibitory profile towards Akt1. Mechanistic studies revealed that compound **5h** induced apoptotic cell death in MCF‐7 cells, disrupted mitochondrial membrane potential, and caused high levels of caspase‐3 activation. Furthermore, molecular docking and molecular dynamics simulations showed that the compound formed stable interactions in the Akt1 and PI3K active sites, supporting the experimental results. The findings indicate that compound **5h** is a promising anticancer candidate that targets the PI3K/Akt signaling pathway.

## Graphical abstract

Novel benzothiazole‐triazole derivatives were designed and synthesized as potential anticancer agents targeting the PI3K/Akt pathway. Compound **5h** exhibited potent Akt1 inhibition, induced apoptosis and mitochondrial dysfunction in MCF‐7 cells, and demonstrated stable binding in molecular docking and dynamics studies, highlighting its promise for breast cancer therapy.

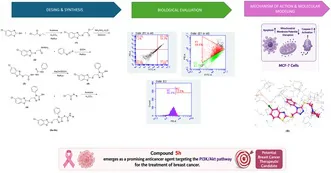

## 1 Introduction

Cancer is a leading cause of death worldwide and is a complex disease characterized by numerous cancer‐associated genes identified in humans. Breast cancer (BC) has been a leading cause of cancer‐related morbidity and mortality among women in most countries for many years (Zhang et al. 2025).

Treatment includes surgical methods (lumpectomy and mastectomy) and adjuvant therapy (chemotherapy, radiotherapy, hormone therapy, targeted therapies, and immunotherapy) (Yuan et al. 2023). In advanced stages, where the tumor has metastasized, chemotherapy can help alleviate cancer‐related symptoms (Anand et al. 2023).

Apoptosis is programmed cell death, a normal and controlled process that supports cell growth and death. Inducing apoptosis is an important strategy to control the proliferation of breast cancer cells (Yuan et al. 2022). However, abnormal apoptosis and uncontrolled growth can also cause cancer cells to multiply rapidly (D'Arcy 2019). Among the many proteins involved in regulating apoptosis, some play a central role in the balance between cell survival and death; Akt (protein kinase B, EC 2.7.11.1) is one of them (Du and Tsichlis 2005; Afzal et al. 2026). Akt is an enzyme that is involved in various signaling pathways, including PI3K/Akt/mTOR, which control cell growth, proliferation, and metabolism (Hinz and Jücker 2019). Akt1 inhibits apoptosis by phosphorylating its targets, stimulates cell division, and promotes cellular resistance to stress; these properties make it a central target in tumor development (Afzal et al. 2026).

Akt, a key component of the signaling pathway, exists in three different isoforms: Akt1, Akt2, and Akt3. Akt hyperactivation has been associated with cancer cells evading immune system control (Liu et al. 2009) and is dependent on PI3K (Engelman 2009). It supports tumor cell growth, proliferation, and survival by modulating the cell cycle through the signaling cascade (Xue et al. 2015). As it regulates key features of cancer such as tumor growth, metastasis, and angiogenesis (Islam et al. 2023) significant efforts have been made to develop therapies targeting Akt signaling in BC (He et al. 2021; Castaneda et al. 2010). Furthermore, as the PI3K/Akt signaling pathway is frequently disrupted in up to 70% of human BCs (Araki and Miyoshi 2018) and Akt upregulation in cancer is generally associated with poor prognosis (Hernandez‐Aya and Gonzalez‐Angulo 2011), Akt has been considered a reasonable target for BC treatment. However, the fact that the different isoforms—Akt1, Akt2, and Akt3—have non‐interchangeable effects on tumor formation makes inhibition targeting all Akt isoforms in BC inappropriate. Studies have reported that Akt1 plays a tumor‐initiating role, while Akt2 is primarily responsible for tumor progression and metastasis. Evidence regarding the role of Akt3 in regulating key features of BC is limited. Current research shows that inhibition of the PI3K/Akt pathway enhances tumor immune surveillance by blocking immunosuppressive pathways. In BC, dysregulation of this pathway significantly contributes to the development of resistance to chemotherapeutic drugs and targeted therapies. Therefore, numerous clinical studies have reported that inhibitors targeting both enzymes are highly effective therapeutic agents, offering advantages in overcoming resistance and improving disease prognosis (López‐Knowles et al. 2010; Pérez‐Tenorio et al. 2002; Dotolo et al. 2021; Spencer et al. 2014).

The SAR properties of Akt1 inhibitors are as follows: π–π stacking interaction with an aromatic or heteroaromatic chain, which is essential for binding to the Trp80 amino acid; various interactions beginning with HBDs to the Tyr272 residue; and other HBDs and HBAs for the Asp274 amino acid. Additional hydrophobic interactions involving the Leu210, Leu264, and Ile290 residues can also be observed (Maurya and Vinayak 2017; Helwa et al. 2020).

Phosphoinositide 3‐kinases (PI3Ks) (EC 2.7.1.154, EC 2.7.1.154) are a family of lipid kinases that integrate signals from growth factors, cytokines, and other environmental stimuli, converting them into intracellular signals that regulate multiple signaling pathways. These pathways control many physiological functions and cellular processes, including cell proliferation, growth, survival, motility, and metabolism (Mili et al. 2024). It manages cellular processes to maintain the balance between cell survival and apoptosis. The frequent occurrence of activating changes in PI3K in various cancer types has made this class of enzymes a primary drug target for anticancer therapy (Reheim et al. 2025).

Study of various PI3K inhibitors and their binding patterns has revealed four key structure–activity relationship (SAR) properties. A group such as a morpholine ring is required as a hydrogen bond acceptor (HBA) to bind to the key amino acid Val851 in the hinge region. Any heteroaromatic structure (mono‐ or fused heterocyclic ring) can serve as the central core. For side chain interactions, an aryl substituent or lipophilic side chain containing a hydrogen bond donor (HBD) or HBA at the meta position relative to the morpholine group interacts with the amino acids Tyr836, Asp810, or Lys802. In the PI3K binding site, some inhibitors show expansion towards the solvent‐exposed region. This expansion may create additional hydrogen bond interactions with Lys and Ala or improve the pharmacokinetic properties of the developed derivatives (Zhong and Goodwin 2024).

Heterocyclic‐based compounds are valuable scaffolds in anticancer drug discovery because they are highly effective in targeting various metabolic pathways and cellular processes involved in cancer pathology. The benzothiazole ring system is a critical pharmacophore in drug discovery due to its antitumor, antimetastatic, and selective cytotoxic properties (Irfan et al. 2020). Literature reports that benzothiazole derivatives exhibit high affinity in resistant BC cell lines (MCF‐7, MDA‐MB‐231) and induce apoptosis by arresting the cell cycle (Irfan et al. 2020; Barbarossa et al. 2023). In addition, the 1,2,4‐triazole core is known for its high bioavailability, low toxicity, and strong interaction capacity with amino acids in enzyme active sites, especially due to its HBD and HBA abilities (Khasawneh et al. 2025; Akhter et al. 2023). Combining these two strong backbones aims to improve both the pharmacokinetic profile and the binding affinity to target enzymes (PI3K and Akt1) because of the molecular hybridization strategy. In similar studies in the literature, benzothiazole‐triazole hybrid structures have been reported to show strong inhibition of the EGFR/PI3K/Akt pathway and significantly induce apoptosis (Alsehli et al. 2025; Rezki et al. 2020; Aouad et al. 2018).

In the compounds synthesized in this study, the benzothiazole backbone replaced the heteroaromatic units in the core structure of PI3K inhibitors such as PI‐103. The aim was to block the ATP‐binding site of the PI3K enzyme by interacting with amino acid residues in the hinge region of the benzothiazole, using its sulfur and nitrogen atoms. The triazole ring, positioned between the benzothiazole and the side chains, was designed not only as a linker but also as a pharmacophore capable of hydrogen bonding with key amino acids such as Val851 (PI3K) or Tyr272 (Akt1) in the active site. The hydrophobic character of the benzothiazole ring facilitates π–π stacking interactions with Trp80 in the active site of the Akt1 enzyme. Furthermore, substituents (e.g., aryl groups) added to the triazole ring were intended to extend to the solvent‐exposed regions of the enzyme, optimizing its pharmacokinetic properties and target selectivity.

**FIGURE 1:**
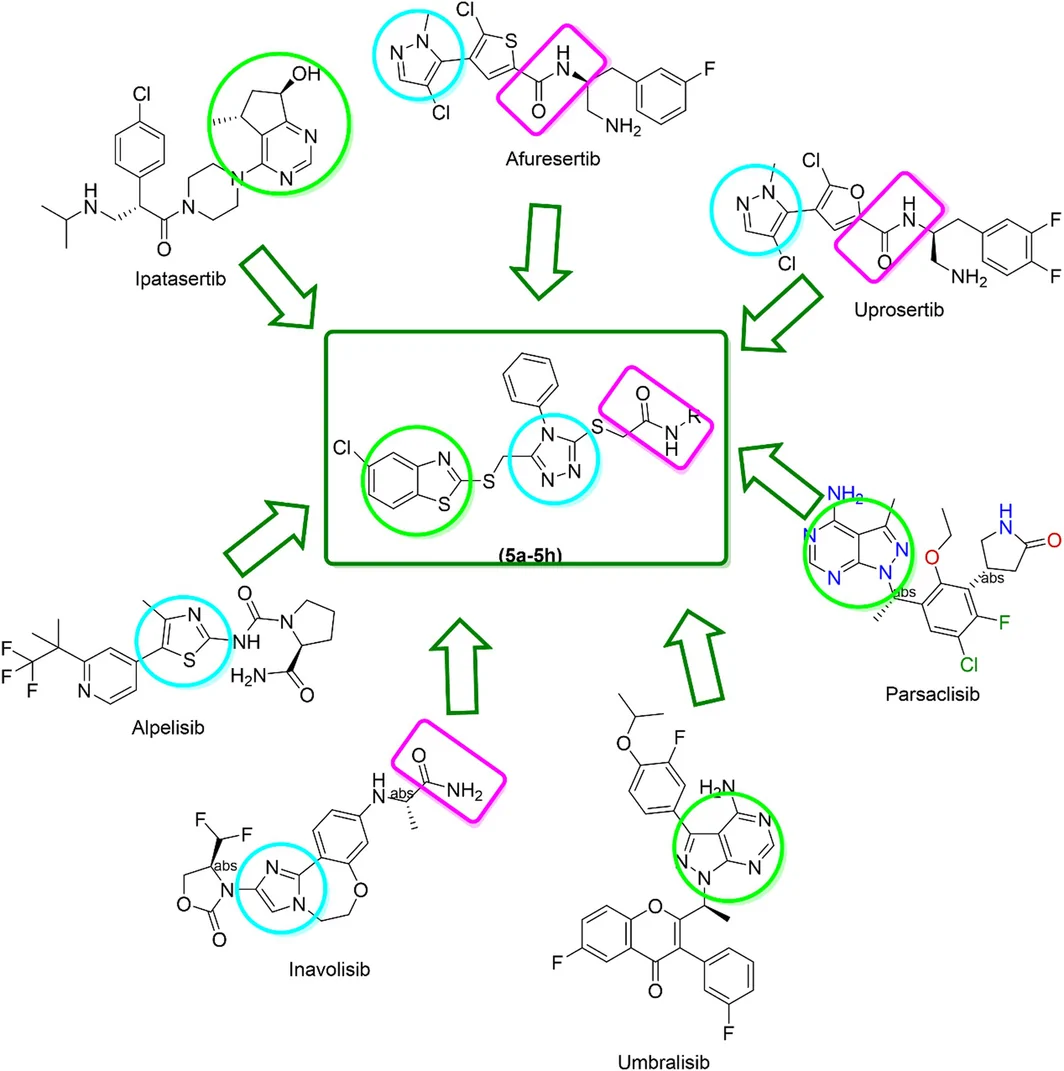
Chemical structures of representative AKT and PI3K inhibitors and the designed target compounds.

The chemical structures of some current AKT and PI3K inhibitors and the target compounds designed in this study are shown in Figure 1. Common structural motifs and similar pharmacophore regions are marked using geometric shapes of the same color to facilitate comparison.

## 2 Material and Methods

### 2.1 Chemistry

All reagents were purchased from commercial suppliers and no purification was performed before use. Characterization of the compounds was carried out using a ^1^H‐NMR DPX 400 FT‐NMR spectrometer and a ^13^C‐NMR DPX 100 MHz spectrometer (Bruker Bioscience, USA). Mass spectrometers were recorded using the ESI technique and a LCMS‐IT‐TOF (Shimadzu, Kyoto, Japan) instrument. Purity analyses were performed using the PDA detector of the LCMS‐IT‐TOF instrument (Figures S1–S32).

#### 2.1.1 Synthesis of Ethyl 2‐[(5‐chlorobenzothiazol‐2‐yl)thio]acetate (**1**)

A mixture of 5‐chlorobenzo[*d*]thiazole‐2‐thiol (9.935 g, 0.0492 mol) and ethyl chloroacetate (6.0365 g, 0.0492 mol) was refluxed for **5h** in acetone using potassium carbonate (8.160 g, 0.0590 mol) as a catalyst. The progress of the reaction was monitored by TLC. Upon completion, acetone was removed. The resulting product was rinsed with water, dried, and collected via filtration.

#### 2.1.2 Synthesis of 2‐[(5‐Chlorobenzothiazol‐2‐yl)thio]acetohydrazide (**2**)

Ethyl 2‐[(5‐chlorobenzothiazol‐2‐yl)thio]acetate (8.941 g, 0.0326 mol), an excess of hydrazine hydrate in ethanol was added in portions. The mixture was then stirred at room temperature for **5h**.

#### 2.1.3 Synthesis of 1‐{[(5‐Chlorobenzo[d]thiazol‐2‐yl)sulfanyl]acetyl}‐4‐phenylthiosemicarbazide (**3**)

A mixture of compound 2 (8.047 g, 0.0221 mol) and phenyl isothiocyanate was dissolved in ethanol (20 mL). The mixture is stirred under reflux for **6h**.

#### 2.1.4 Synthesis of 5‐{[(5‐Chlorobenzo[d]thiazol‐2‐yl)sulfanyl]methyl}‐4‐phenyl‐2,4‐dihydro‐3H‐1,2,4‐triazole‐3‐thione (**4**)

A mixture of compound 4 (7.242 g, 0.0178 mol) was added to EtOH (20 mL) containing sodium hydroxide and refluxed for **5h**. At the end of the reaction, the reaction mixture was dropped into ice water and acidified to pH 4–5 with 20% HCl.

#### 2.1.5 Synthesis of the Target Compounds

5‐{[(5‐Chlorobenzo[d]thiazol‐2‐yl)sulfanyl]methyl}‐4‐phenyl‐2,4‐dihydro‐3*H*‐1,2,4‐triazole‐3‐thione (0.3 g, 0.0008 mol) was reacted with eight different N‐substituted chloroacetamide derivatives (0.0008 mol) in acetone in the presence of potassium carbonate (0.133 g, 0.001 mol). The mixture was heated under reflux, and the reaction was monitored by TLC. The resulting solid products were filtered, washed with ethanol, and dried.

##### 2.1.5.1 2‐((5‐(((5‐Chlorobenzo[d]thiazol‐2‐yl)thio)methyl)‐4‐phenyl‐4H‐1,2,4‐triazol‐3‐yl)thio)‐N‐methylacetamide (**5a**)

Yield: %71. ^1^H‐NMR (400 MHz, DMSO‐*d*_6_): δ = 2.59 (3H, s, ‐CH_3_), 3.90 (2H, s, ‐CH_2_‐), 4.69 (2H, s, ‐CH_2_‐), 7.41 (1H, d, *J* = 6.4 Hz, Ar‐H), 7.55–7.58 (6H, m, 4H, Ar‐H), 7.84 (1H, s, Ar‐H), 8.02 (1H, d, *J* = 6.3 Hz, Ar‐H), 8.16 (1H, s, Ar‐H). ^13^C‐NMR (100 MHz, DMSO‐*d*_6_): δ = 26.4, 27.0, 36.2, 121.2, 123.7, 125.1, 127.7, 130.4, 130.7, 131,7, 132.9, 134.1, 151.7, 151.9, 153.5, 167.3, 167.8 HRMS (m/z): [M + H] ^+^ calc for C_19_H_16_N_5_OS_3_Cl: 462.0278; found: 462.0283 (100%).

##### 2.1.5.2 2‐((5‐(((5‐Chlorobenzo[d]Thiazol‐2‐Yl)thio)methyl)‐4‐Phenyl‐4H‐1,2,4‐Triazol‐3‐Yl)thio)‐N‐Isopropylacetamide (**5b**)

Yield: %82.^1^H‐NMR (400 MHz, DMSO‐*d*_6_): δ = 1.1–1.3 (6H, s, ‐CH_3_), 3.76–3.78 (1H, m, ‐CH), 3.88 (2H, s, ‐CH_2_‐), 4.68 (2H, s, ‐CH_2_‐), 7.42 (1H, d, *J* = 6.2 Hz, Ar‐H), 7.55–7.58 (4H, m, Ar‐H), 7.85 (1H, s, Ar‐H), 8.03 (1H, d, *J* = 6.2 Hz, Ar‐H), 8.10–8.12 (1H, m, Ar‐H). ^13^C‐NMR (100 MHz, DMSO‐*d*_6_): δ = 22.7, 27.1, 36.7, 41.4, 121.2, 123.8, 125.2, 127.8, 130.4, 130.6, 131.7, 132.9, 134.1, 151.7, 151.8, 153.5, 165.8, 167.8 HRMS (m/z): [M + H] ^+^ calc for C_21_H_20_N_5_OS_3_Cl: 490.0591; found: 490.0582 (96.12%).

##### 2.1.5.3 2‐((5‐(((5‐Chlorobenzo[d]thiazol‐2‐yl)thio)methyl)‐4‐phenyl‐4H‐1,2,4‐triazol‐3‐yl)thio)acetamide (**5c**)

Yield: %77.^1^H‐NMR (400 MHz, DMSO‐*d*_6_): δ = 3.92 (2H, s, ‐CH_2_‐), 4.69 (2H, s, ‐CH_2_‐), 7.25 (1H, br.s.), 7.41 (1H, d, *J* = 6.3 Hz, Ar‐H), 7.55–7.58 (5H, m, Ar‐H), 7.68 (1H, s, Ar‐H), 7.84 (1H, s, Ar‐H), 8.02 (1H, d, *J* = 6.3 Hz, Ar‐H). ^13^C‐NMR (100 MHz, DMSO‐*d*_6_): δ = 27.0, 36.4, 121.2, 123.8, 125.2, 127.7, 130.4, 130.7, 131.7, 132.9, 134.1, 151.8, 151.9, 153.5, 167.8, 168.9 HRMS (m/z): [M + H] ^+^ calc for C_18_H_14_N_5_OS_3_Cl: 448.0122; found: 448.0122 (100%).

##### 2.1.5.4 2‐((5‐(((5‐Chlorobenzo[d]thiazol‐2‐yl)thio)methyl)‐4‐phenyl‐4H‐1,2,4‐triazol‐3‐yl)thio)‐N‐methoxy‐N‐methylacetamide (**5d**)

Yield: %79.^1^H‐NMR (400 MHz, DMSO‐*d*_6_): δ = 3.11 (3H, s, ‐CH_3_), 3.71 (3H, s, ‐CH_3_), 4.28 (2H, s, ‐CH_2_‐), 4.69 (2H, s, ‐CH_2_‐), 7.41 (1H, d, *J* = 6.3 Hz, Ar‐H), 7.55–7.57 (5H, m, Ar‐H), 7.84 (1H, s, Ar‐H), 8.02 (1H, d, *J* = 6.3 Hz, Ar‐H). ^13^C‐NMR (100 MHz, DMSO‐*d*_6_): δ = 27.0, 32.6, 35.1, 61.7, 117.4, 121.2, 123.8, 125.2, 127.7, 130.4, 130.7, 131.7, 132.9, 134.1, 151.5, 151.9, 153.5, 167.8 HRMS (m/z): [M + H] ^+^ calc for C_20_H_18_N_5_O_2_S_3_Cl: 492.0384; found: 492.0378 (100%).

##### 2.1.5.5 2‐((5‐(((5‐Chlorobenzo[d]thiazol‐2‐yl)thio)methyl)‐4‐phenyl‐4H‐1,2,4‐triazol‐3‐yl)thio)‐N‐cyclopropylacetamide (**5e**)

Yield: %85.^1^H‐NMR (400 MHz, DMSO‐*d*_6_): δ = 0.38 (2H, s, Ar‐H), 0.59–0.61 (2H, m, Ar‐H), 2.59 (1H, br.s., Ar‐H), 3.86 (2H, s, ‐CH_2_‐), 4.62 (2H, s, ‐CH_2_‐), 7.42 (1H, d, *J* = 6.2 Hz, Ar‐H), 7.55–7.58 (4H, m, Ar‐H), 7.8 (1H, s, Ar‐H), 8.04 (1H, d, *J* = 6.2 Hz, Ar‐H), 8.30 (1H, s, Ar‐H). ^13^C‐NMR (100 MHz, DMSO‐*d*_6_): δ = 6.1, 23.0, 27.0, 36.4, 121.2, 123.8, 125.2, 127.7, 130.4, 130.7, 131.7, 132.9, 134.1, 151.7, 151.9, 153.5, 167.8, 167.9 HRMS (m/z): [M + H]^+^ calc for C_21_H_18_N_5_OS_3_Cl: 488.0435; found: 488.0430 (100%).

##### 2.1.5.6 2‐((5‐(((5‐Chlorobenzo[d]thiazol‐2‐yl)thio)methyl)‐4‐phenyl‐4H‐1,2,4‐triazol‐3‐yl)thio)‐N,N‐dimethylacetamide (**5f**)

Yield: %83.^1^H‐NMR (400 MHz, DMSO‐*d*_6_): δ = 2.82 (3H, s, ‐CH_3_), 2.99 (3H, s, ‐CH_3_), 4.27 (2H, s, CH_2_), 4.69 (2H, s, ‐CH_2_‐), 7.42 (1H, d, *J* = 6.0 Hz, Ar‐H), 7.55–7.57 (5H, m, Ar‐H), 7.85 (1H, s, Ar‐H), 8.03 (1H, d, *J* = 6.1 Hz, Ar‐H). ^13^C‐NMR (100 MHz, DMSO‐*d*_6_): δ = 27.0, 35.8, 37.1, 37.4, 121.2, 123.8, 125.2, 127.8, 130.4, 130.7, 131.7, 132.9, 134.1, 151.7, 151.8, 153.5, 166.8, 167.8 HRMS (m/z): [M + H] ^+^ calc for C_20_H_18_N_5_OS_3_Cl: 476.0435; found: 476.0440 (100%).

##### 2.1.5.7 2‐((5‐(((5‐Chlorobenzo[d]thiazol‐2‐yl)thio)methyl)‐4‐phenyl‐4H‐1,2,4‐triazol‐3‐yl)thio)‐N,N‐diethylacetamide (**5g**)

Yield: %80.^1^H‐NMR (400 MHz, DMSO‐*d*_6_): δ = 0.98 (3H, t, *J* = 5.0 Hz, ‐CH_3_), 1.12 (3H, t, *J* = 4.9 Hz, CH_3_), 3.23–33.36 (4H, m, ‐CH_2_‐), 4.25 (2H, s, ‐CH_2_‐), 4.69 (2H, s, ‐CH_2_‐), 7.42 (1H, d, *J* = 6.3 Hz, Ar‐H), 7.55–7.59 (5H, m, Ar‐H), 7.84 (1H, s, Ar‐H), 8.02 (1H, d, *J* = 6.3 Hz, Ar‐H). ^13^C‐NMR (100 MHz, DMSO‐*d*_6_): δ = 13.3, 14.4, 27.0, 30.1, 36.9, 42.3, 121.2, 123.7, 125.1, 127.8, 130.4, 130.7, 131.7, 132.9, 134.1, 151.7, 151.8, 153.5, 165.9, 167.8 HRMS (m/z): [M + H] ^+^ calc for C_22_H_22_N_5_OS_3_Cl: 504.0748; found: 504.0745 (100%).

##### 2.1.5.8 2‐((5‐(((5‐Chlorobenzo[d]thiazol‐2‐yl)thio)methyl)‐4‐phenyl‐4H‐1,2,4‐triazol‐3‐yl)thio)‐1‐morpholinoethan‐1‐one (**5h**)

Yield: %88.^1^H‐NMR (400 MHz, DMSO‐*d*_6_): δ = 3.41–3.46 (4H, m, morpholine), 3.52–357 (4H, m, morpholine), 4.26 (2H, s, ‐CH_2_‐), 4.69 (2H, s, ‐CH_2_‐), 7.42 (1H, d, *J* = 6.4 Hz, Ar‐H), 7.55–7.59 (5H, m, Ar‐H), 7.85 (1H, s, Ar‐H), 8.03 (1H, d, *J* = 6.4 Hz, Ar‐H). ^13^C‐NMR (100 MHz, DMSO‐*d*_6_): δ = 27.0, 36.6, 42.4, 46.3, 66.3, 121.2, 123.8, 125.2, 127.8, 130.4, 130.7, 131.7, 132.9, 134.1, 151.5, 151.9, 153.5, 165.8, 167.8 HRMS (m/z): [M + H] ^+^ calc for C_22_H_20_N_5_O_2_S_3_Cl: 518.0540; found: 518.0538 (100%).

### 2.2 Anticancer Activity Studies

#### 2.2.1 Cytotoxicity Studies

The anticancer effects of compounds **5a–5h** against the MCF‐7 cell line were determined using the MTT assay. Imatinib and anastrozole were used as reference drugs in calculating the IC_50_ values. Both the synthesized compounds and the standards were tested and evaluated at various concentrations ranging from 1000 to 0.316 μM. Analyses were performed using fluorometric measurements (Osmaniye, Levent, Ardıç, et al. 2018; Osmaniye, Levent, Karaduman, et al. 2018; Osmaniye, Korkut Çelikateş, et al. 2021).

#### 2.2.2 In Vitro Enzyme Studies

In vitro aromatase inhibition assays were performed using a commercial kit according to the manufacturer's protocol (Abcam, Aromatase (CYP19A) Inhibitor Screening Kit, fluorometric method) (Abcam n.d.; Osmaniye, Görgülü, et al. 2021). The compounds were dissolved in 2% dimethyl sulfoxide (DMSO) (in buffer) and tested at a minimum of seven different concentrations ranging from 10^−3^ to 10^−9^ M. Letrozole was used as the positive inhibition control. In vitro Akt‐1 inhibition assays were performed using a commercial kit according to the manufacturer's protocol (BPBioscience, 78038) (BPS Bioscience n.d.). The compounds were dissolved in 2% dimethyl sulfoxide (DMSO) (in buffer) and tested at a minimum of seven different concentrations ranging from 10^−3^ to 10^−9^ M. Letrozole was used as the positive inhibition control.

#### 2.2.3 Apoptosis Assessment by Annexin V/PI, MMP, and Caspase‐3 Assays

##### 2.2.3.1 Flow Cytometry Analysis

Apoptosis is programmed cell death, a physiological process in healthy organisms that removes cells which have completed their function. During apoptosis, phosphatidylserine (PS), normally located on the inner surface of the cell membrane, translocates to the outer surface. The Annexin V protein included in the kit specifically binds to this externalized PS and is detected using a fluorescent marker such as FITC. However, as Annexin V is not specific to apoptotic cells, counterstains such as propidium iodide (PI) are used to distinguish between apoptotic and necrotic cells. This flow cytometry‐based double staining method enables differentiation of viable, early apoptotic, late apoptotic, and necrotic cell populations by evaluating Annexin V–PS binding together with PI permeability (Yurtdaş‐Kırımlıoğlu et al. 2020; Yurttas et al. 2017; ABP Biosciences n.d.).

In summary, MCF‐7 cells were plated in six‐well plates following the manufacturer's protocol and exposed to IC_50_ concentrations of the compounds for 24 h. After incubation, cell suspensions were collected by centrifugation at 1200 rpm for 5 min, and the cell pellets were washed twice with cold phosphate‐buffered saline (PBS). The cells were then transferred to buffer solutions according to the kit protocol. At least 10,000 cells were analyzed for each sample, and the proportions of different cell populations were evaluated by comparison with a control group using appropriate statistical methods.

##### 2.2.3.2 Analysis of Mitochondrial Membrane Potential (MMP) by Flow Cytometry

To evaluate changes in mitochondrial membrane potential (MMP), the BD MitoScreen Mitochondrial Membrane Potential Detection JC‐1 kit (BD Biosciences) was used. MCF‐7 cells were seeded into 25 mL culture flasks under appropriate conditions and incubated for 24 h in an incubator containing 5% CO_2_. After cell adhesion, the **5h** compound and the reference drug were applied to the cells at IC_50_ concentrations, followed by a further 24‐h incubation.

At the end of the incubation, the cells were collected and centrifuged according to the kit protocol. After removing the supernatant, JC‐1 dye was added to the cell pellet and incubated at 37°C for 10–15 min. The cells were then washed twice with the washing solution and prepared for analysis. MMP changes were assessed using CytoFLEX flow cytometry (Beckman Coulter Life Sciences), and the data were analyzed using CytoExpert software (version 2.2.0.97) (Ali et al. 2023). Two drugs with different mechanisms of action were selected as reference drugs. One of them is the aromatase inhibitor anastrazole, and the other is the kinase inhibitor imatinib.

##### 2.2.3.3 Caspase‐3 Activation Analysis

Caspase‐3 activation was analyzed by flow cytometry to assess enzymatic activity associated with apoptosis. MCF‐7 cells were treated with the **5h** compound and the reference drug at IC_50_ concentrations for 24 h. After incubation, cells were collected and stained according to the manufacturer's protocol for caspase‐3 detection. Caspase‐3 positive and negative cell populations were determined by flow cytometry and expressed as percentage distributions.

### 2.3 Molecular Docking

Molecular docking studies were performed using the Glide module in Schrödinger Maestro (version 2020) software. Crystal structures obtained from the Protein Data Bank were prepared for docking studies using the Protein Preparation Wizard module. This included adding missing hydrogen atoms, correcting bond arrangements, assigning appropriate protonation states, removing water molecules far from the active site, optimizing the hydrogen bond network, and subjecting protein structures to energy minimization using the OPLS4 force field.

Ligand structures were prepared and energy minimization performed under physiological pH (7.0 ± 1.0) conditions using the LigPrep module. The receptor grid was created centered on the co‐crystallized ligand in the crystal structure. Molecular docking calculations were performed using the Glide Standard Precision (SP) algorithm with default parameters. For each compound, the binding pose with the lowest GlideScore was selected for further analysis.

Molecular docking studies were conducted to investigate the binding interactions between the compound **5h** and AKT‐1 and PI3K, and to elucidate potential mechanisms of anticancer activity. Docking simulations were carried out using Maestro software (Schrödinger 2020a, 2020b, 2020c).

### 2.4 Molecular Dynamics Simulations

Molecular dynamics simulations (MDS) were performed using Schrödinger Suite software packages to determine the stability of the selected samples with the target enzyme and the binding modes of the interactions. The detailed protocol and procedural steps for the simulations performed using Desmond (Schrödinger's MD module) and Maestro (Schrödinger's graphical user interface) have been previously reported by our system (Schrödinger 2020b; Liu et al. 2018; M.D.I. Tools 2020; Sureshkumar et al. 2018; Humphreys et al. 1994; Hoover 1985; Martyna et al. 1994; Essmann et al. 1995; Uslu et al. 2025; Osmaniye et al. 2024).

## 3 Results and Discussion

### 3.1 Chemistry

**SCHEME 1:**
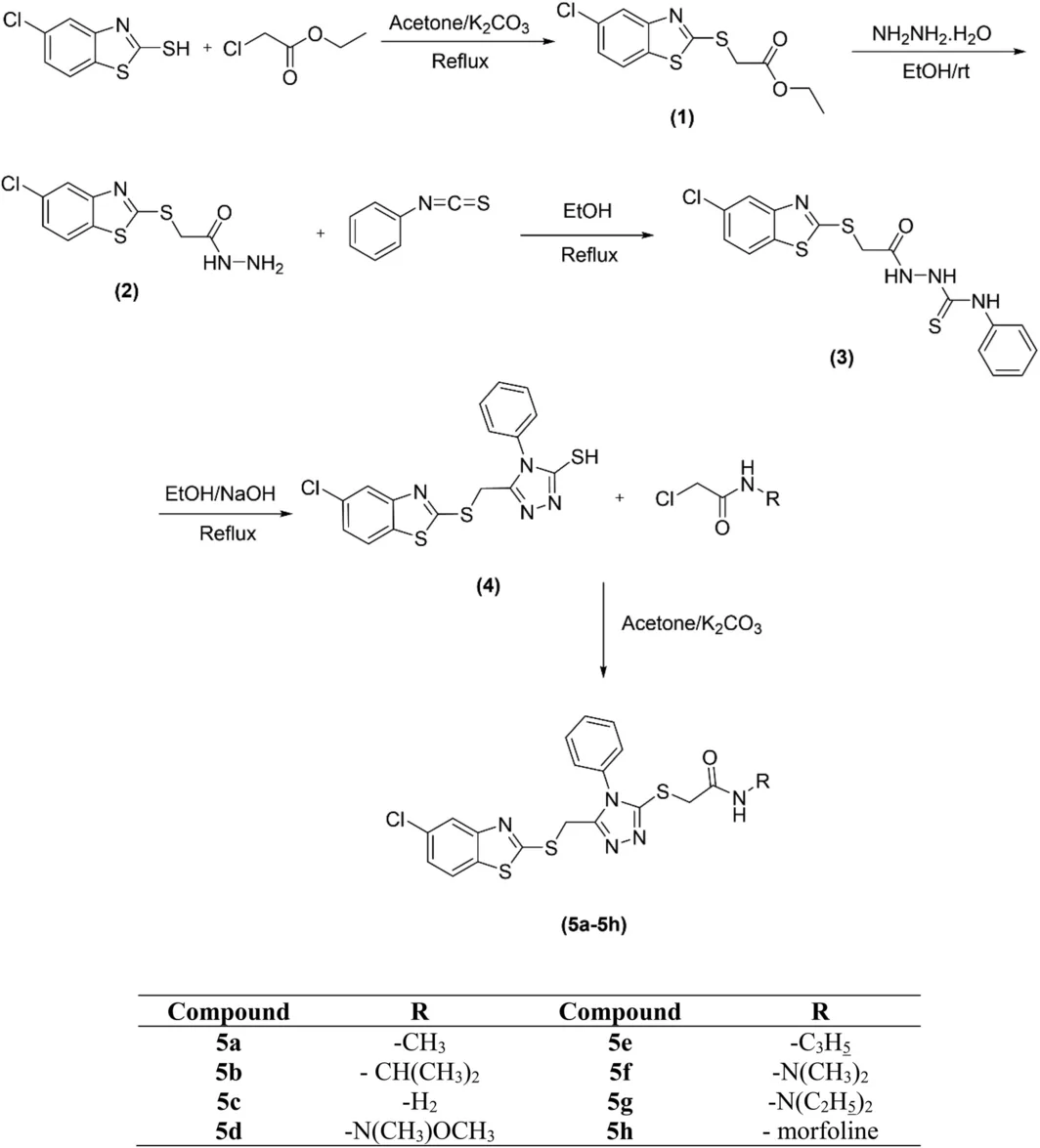
Synthesis pathway for obtained compounds **5a–5h**.

Ethyl 2‐[(5‐chlorobenzothiazol‐2‐yl)thio]acetate **(1)** was prepared by heating 5‐chlorobenzo[*d*]thiazole‐2‐thiol and ethyl chloroacetate in acetone in the presence of potassium carbonate under reflux for **5h**. After the reaction, the acetone was evaporated, the residue was washed with water, and the product was filtered. The resulting ester derivative (1) was then reacted with hydrazine hydrate in ethanol, added in portions at room temperature. The progress of the reaction was monitored by thin‐layer chromatography (TLC). After 12 h, the precipitate formed was filtered and dried to obtain the hydrazide derivative 2‐[(5‐chlorobenzothiazol‐2‐yl)thio]acetohydrazide (**2**). Compound (**2**) was mixed with phenyl isothiocyanate in absolute ethanol and heated under reflux for 6 h. Upon completion of the reaction, the resulting solid product, 1‐{[(5‐chlorobenzo[d]thiazol‐2‐yl)sulfanyl]acetyl}‐4‐phenylthiosemicarbazide (**3**), was filtered to obtain the intermediate. Compound (**3**) was subjected to cyclisation by heating in ethanol containing sodium hydroxide under reflux for **5h**. The reaction, monitored by TLC, was terminated with 20% hydrochloric acid. The product was filtered to obtain the triazole derivative 5‐{[(5‐chlorobenzo[*d*]thiazol‐2‐yl)sulfanyl]methyl}‐4‐phenyl‐2,4‐dihydro‐3H‐1,2,4‐triazole‐3‐thione (**4**). For the synthesis of the target compounds (**5a–5h**), compound (**4**) was reacted with eight different *N*‐substituted chloroacetamide derivatives in acetone in the presence of potassium carbonate. The mixture was heated under reflux, and the reaction was monitored by TLC. The resulting solid products were filtered, washed with ethanol and dried. As shown in Scheme 1, eight different benzothiazole/triazole derivative compounds were obtained.

The structures of the synthesized compounds (**5a–5h**) were confirmed using NMR analyses (^1^H‐NMR, ^13^C‐NMR). The results confirmed the presence of key functional groups, including benzothiazole, triazole, and phenyl rings, *S*‐thiocarbamate within the molecular framework.

These spectroscopic findings confirm the successful synthesis of compounds **5a–5h**, and the proposed structures are also supported by NMR data. Detailed spectroscopic analyses for each compound are presented in Figures S1–S32.

### 3.2 Anticancer Activity Studies

#### 3.2.1 Cytotoxicity Studies

Cytotoxicity assays performed on the MCF‐7 human BC cell line were evaluated following a 24‐h incubation period. In the assessment of anticancer activity, the absence of significant toxicity toward healthy cells is considered a critical criterion. Therefore, mouse embryonic fibroblast cells (NIH/3 T3) were employed as a healthy cell model in this study. The results obtained are summarized in Table 1.

**TABLE 1:** Cytotoxic activity of the compounds (μM) against MCF‐7, MDA‐MB‐231, and NIH3T3 cell lines (**5a–5h**)^a^.

Compounds	MCF‐7	MDA‐MB‐231	NIH3T3	SI (MCF‐7/NIH3T3)	SI (MCF‐7/MDA‐MB‐231)
**5a**	25.664 ± 0.896	33.693 ± 0.536	49.904 ± 1.269	1.944	1.313
**5b**	14.739 ± 0.156	> 100	34.676 ± 1.036	2.352	> 6.784
**5c**	52.020 ± 2.009	> 100	18.164 ± 0.486	0.349	> 1.922
**5d**	51.968 ± 1.362	90.668 ± 1.023	76.284 ± 0.693	1.468	1.745
**5e**	32.609 ± 1.896	> 100	80.286 ± 2.364	2.462	> 3.066
**5f**	14.133 ± 0.463	33.086 ± 0.896	22.349 ± 0.665	1.581	2.341
**5g**	28.830 ± 1.058	61.221 ± 1.146	60.540 ± 1.963	2.099	2.123
**5h**	10.186 ± 0.123	24.411 ± 0.639	60.974 ± 1.658	5.986	2.396

^a^ The test results were reported as the mean values of four independent assays.

Analysis of the data revealed that the IC_50_ values of the synthesized compounds ranged from 10.186 to 52.020 μM, indicating that all compounds exhibited varying degrees of anticancer activity against BC cells. Among the tested compounds, **5b**, **5f**, and **5h** demonstrated the most pronounced cytotoxic effects. These compounds exhibited the highest activities, with IC_50_ values of 10.186, 14.133, and 14.739 μM, respectively.

The higher antiproliferative activity observed in MCF‐7 cells compared to MDA‐MB‐231 cells may be attributed to the different molecular characteristics of these breast cancer subtypes. MCF‐7 cells are estrogen receptor positive and often contain alterations leading to activation of the PI3K/Akt signaling pathway, making them more dependent on this pathway for survival. In contrast, MDA‐MB‐231 cells represent a triple‐negative, highly invasive phenotype characterized by greater genetic heterogeneity, epithelial‐mesenchymal transition, and intrinsic drug resistance, which may reduce their sensitivity to Akt‐targeted compounds.

The cytotoxic activities and selectivity of the synthesized compounds were found to be significantly affected by the structure of the R group. Among the compound series, compound **5h**, which carries a morpholine group, showed the lowest IC_50_ value (10.186 ± 0.123 μM) and the highest selectivity index (SI = 5.986) in the MCF‐7 cell line. This result indicates that the morpholine ring both enhances anticancer activity and improves selectivity towards tumor cells by reducing toxicity towards healthy NIH3T3 cells. The ability of the tertiary amine nitrogen and ether oxygen in the morpholine ring to establish additional polar interactions with the target protein and optimize the hydrophilic/lipophilic balance of the compound may have contributed to this activity. Although compounds **5f** and **5g**, which contain open‐chain tertiary amine groups, also showed activity, the selectivity index remained low (SI = 1.581), especially due to the higher toxicity of 5f towards healthy cells. Similarly, derivatives bearing methyl (**5a**), isopropyl (**5b**), and allyl (**5e**) groups showed moderate activity but did not exhibit as strong and selective an effect as the morpholine derivative. The fact that compound **5c**, bearing hydrogen, showed the lowest activity and had a selectivity index below 1 (SI = 0.349) indicates that a suitable substituent is critically important for both biological activity and selectivity. The dimethylmethoxyamino group in compound **5d**, while reducing toxicity to healthy cells, did not significantly increase anticancer activity. Overall, it was concluded that the morpholine ring is the most suitable structural modification in this series that increases both biological activity and selectivity against cancer cells.

The selectivity index (SI) values were calculated as 2.352 for compound **5b**, 1.581 for compound **5f**, and 5.986 for compound **5h**. Considering all MTT assay results collectively, compound **5h** emerged as the most promising candidate for further biological investigations due to its potent anticancer activity, relatively low IC_50_ value, and favorable selectivity profile.

#### 3.2.2 In Vitro Enzyme Studies

The results of inhibition studies performed on Akt1 and aromatase enzymes are presented in Table 2. Among the tested compounds, **5h** showed a significant inhibitory effect on the Akt1 enzyme and was determined to be the most active compound with an IC_50_ value of 0.209 ± 0.011 μM. This value is quite close to the activity of the reference Akt inhibitor A‐674563 (IC_50_ = 0.18 ± 0.002 μM), indicating that **5h** is a strong inhibitor of Akt1. In contrast, the IC_50_ value of **5h** on the aromatase enzyme was found to be above 10 μM, and no significant aromatase inhibition was observed. Similarly, compounds **5b** and **5f** also exhibited low activity on both enzymes. The compounds included in the kit were used as reference compounds.

**TABLE 2:** IC_50_ values (μM) of compounds against Akt1 and aromatase enzymes^a^.

Compounds	Aromatase IC_50_ (μM)	AKT‐1 IC_50_ (μM)
**5b**	> 100	> 1
**5f**	> 10	> 1
**5h**	> 10	0.209 ± 0.011
**A‐674563**	—	0.18 ± 0.002
**Letrozole**	0.026 ± 0.001	—

^a^ Test results are expressed as averages of analyses performed in sets of four.

#### 3.2.3 Apoptosis Assessment by Annexin V/PI, MMP, and Caspase‐3 Assays

**FIGURE 2:**
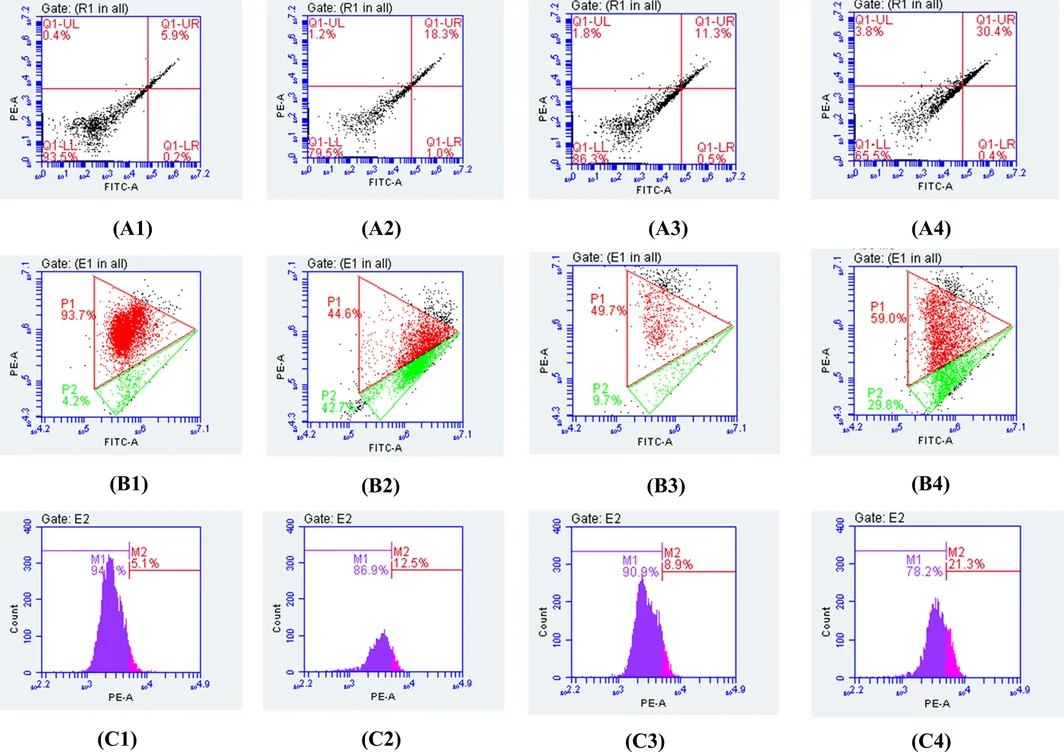
Flow cytometric evaluation of apoptosis‐related parameters in MCF‐7 cells. (A1) Typical Annexin V‐FITC/PI quadrant analysis for growth control, (A2) Typical Annexin V‐FITC/PI quadrant analysis for growth control for compound **5h**, (A3) Typical Annexin V‐FITC/PI quadrant analysis for growth control for compound imatinib, (A4) Typical Annexin V‐FITC/PI quadrant analysis for growth control for compound anastrozole; (B1) effects of compounds on mitochondrial membrane potential (MMP) for growth control, (B2) effects of compounds on mitochondrial membrane potential (MMP) for growth compound **5h**, (B3) effects of compounds on mitochondrial membrane potential (MMP) for imatinib, (B4) effects of compounds on mitochondrial membrane potential (MMP) for anastrozole, and (C1) caspase‐3 activity determined by flow cytometric quadrant analysis for growth control, (C2) caspase‐3 activity determined by flow cytometric quadrant analysis for compound **5h**, (C3) caspase‐3 activity determined by flow cytometric quadrant analysis for imatinib, (C4) caspase‐3 activity determined by flow cytometric quadrant analysis for anastrozole.

Apoptosis is a programmed cell death mechanism that occurs in most tissues and eliminates damaged, abnormal, or dysfunctional cells resulting from cellular stress. This process plays a crucial role in preventing potential pathological conditions and maintaining tissue homeostasis (Carneiro and El‐Deiry 2020; Singh et al. 2018). Unlike necrosis, which occurs because of acute cell damage, apoptosis takes place through tightly regulated molecular mechanisms and is critical for maintaining tissue development and integrity in multicellular organisms. In the apoptotic process, cells shrink and are systematically removed from the environment, while necrosis generally results in uncontrolled cell death, causing an inflammatory response in surrounding tissues (Xu et al. 2019). Disruptions in apoptotic mechanisms at the genetic or metabolic level can play a role in the development of many diseases, including neurodegenerative diseases and cancer (Pistritto et al. 2016). Therefore, apoptosis is critically important in tumor formation, progression, and metastasis processes. In this study, flow cytometry analyses were performed using Annexin V and propidium iodide (PI) staining to evaluate the apoptotic effects of compounds exhibiting potent and selective cytotoxic activity against BC cells. It was determined whether the most effective compound, **5h**, inhibited cells through apoptosis or necrosis, and the results are presented as percentages in Table 3 and as diagrams for the compound and reference drug in Figure 2 (A1)–Figure 2 (A4). The results of the compounds applied at IC_50_ doses were evaluated after 24 h of incubation. Annexin V‐FITC/PI flow cytometry results showed that the **5h** compound produced significant apoptotic activity in MCF‐7 cells. The total apoptotic cell ratio (Q2 + Q4) was determined to be 19.3% for **5h** (Figure 2 (A2)), which was higher than that of the anastrozole (11.8%) (Figure 2 (A3)) and positive control (6.1%) (Figure 2 (A1)) groups. However, it was lower than the 30.8% total apoptotic cell ratio observed in the imatinib group (Figure 2 (A4)). In cells treated with **5h**, late apoptotic cells (Q2, 18.3%) constituted most of the apoptotic population, while the early apoptotic cell ratio (Q4) was determined to be 1.0%. This indicates that a significant portion of the cells reached the advanced stages of the apoptotic process at the end of the 24‐h incubation period. Furthermore, the fact that the necrotic cell rate was only 1.2% in the **5h** group suggests that the observed cytotoxic effect is largely mediated by apoptosis and that necrotic cell death remains limited. The live cell rate (Q3) was determined to be 79.5%, which is lower than the anastrozole group (86.3%) but higher than the imatinib group (65.5%). Overall, it can be said that the **5h** compound significantly induced apoptotic cell death in MCF‐7 cells and that its anticancer effect is significantly related to the apoptosis mechanism.

**TABLE 3:** Flow cytometric evaluation of apoptosis‐related parameters^a^.

Compounds	Annexin V FITC/propidium iodide flow cytometry				
	Q1	Q2	Q3	Q4	Q2 + Q4
**Positive Control**	0.4	5.9	93.5	0.2	6.1
**5h**	1.2	18.3	79.5	1.0	19.3
**Anastrazole**	1.8	11.3	86.3	0.5	11.8
**Imatinib**	3.8	30.4	65.5	0.4	30.8

Compounds	Mitochondrial membrane potential	
	Polarization %	Depolarization %
**Positive Control**	93.7	4.2
**5h**	44.6	42.7
**Anastrazole**	49.7	9.7
**Imatinib**	59.0	29.8

Compounds	Caspase‐3 activity	
	% Caspase‐3 positive (+) cells	% Caspase‐3 negative (−) cells
**Positive Control**	94.5	5.1
**5h**	86.9	12.5
**Anastrazole**	90.9	8.9
**Imatinib**	78.2	21.3

^a^ Q1: necrotic cells, Q2: late apoptotic cells, Q3: viable cells, Q4: early apoptotic cells, Q2 + Q4: early and late apoptotic cells. MCF7 cells were cultured for 24 h in medium with compound **5h** and reference drug at IC_50_ values. At least 10.000 cells were analyzed per sample, and quadrant analysis was performed.

Changes in mitochondrial membrane potential (MMP), considered one of the key indicators of apoptotic cell death, are widely used to assess mitochondrial function in the programmed cell death process. MMP loss is closely associated with mitochondrial dysfunction and contributes to cellular damage and cell death in pathological conditions such as ischemia and oxidative stress (Bock and Tait 2020). While mitochondrial membrane depolarization is primarily associated with the intrinsic apoptotic‐mediated signaling (Galluzzi et al. 2018).

These mitochondrial changes lead to the release of proapoptotic factors into the cytosol, followed by activation of caspase cascades, thus triggering apoptotic cell death.

In this study, the most active compound, **5h**, was comparatively examined with the reference drug to evaluate its effects on mitochondrial membrane potential. The results obtained are presented in Table 3 and Figure 2 (B1) and Figure 2 (B2).

Mitochondrial membrane potential analyses showed that compound **5h** caused significant mitochondrial dysfunction in MCF‐7 cells. Mitochondrial membrane depolarization is considered one of the early and important indicators of the apoptotic process, suggesting that cell death occurs particularly via the intrinsic apoptotic pathway.

In this study, compound **5h** exhibited a depolarization rate of 42.7% and a polarization rate of 44.6% (Figure 2 (B2)). The obtained depolarization value was found to be higher when compared to anastrozole (9.7%) (Figure 2 (B3)) and imatinib (29.8%) (Figure 2 (B4)). It is particularly noteworthy that the depolarization rate caused by **5h** was approximately 4.4 times higher than that caused by anastrozole and approximately 1.4 times higher than that caused by imatinib. These findings indicate that compound **5h** disrupts mitochondrial membrane integrity, negatively impacting mitochondrial function and strongly activating the apoptotic process. When evaluated together with Annexin V/PI and caspase‐3 results, it is thought that the anticancer effect of **5h** occurs largely via the mitochondria‐mediated intrinsic apoptotic pathway, and that mitochondrial membrane depolarization plays a significant role in this mechanism.

Caspases are proteolytic enzymes that play a critical role in the regulation of programmed cell death. Losses and dysregulations in the expression or activity of these enzymes can contribute to tumor formation and progression. Caspase‐3, one of the executive caspases, is involved in the final stages of the apoptotic process, and many anticancer agents induce apoptosis in tumor cells via caspase‐3 activation. However, excessive or uncontrolled activation of caspase‐3 has been associated with some neurodegenerative diseases and has been reported to support carcinogenesis under certain conditions (Jan and Chaudhry 2019).

In this study, the effects of compound **5h** and the reference drug on caspase‐3 activation were evaluated using flow cytometry. Caspase‐3 positive and negative cell populations were determined and compared as percentage distributions. The findings and their diagrams are presented in Table 3 and Figure 2 (C1)–Figure 2 (C4), respectively. When the results of caspase‐3 activation were examined, it was observed that compound **5h** caused a significant level of caspase‐3 activation in MCF‐7 cells. The percentage of caspase‐3 positive cells in cells treated with **5h** was determined to be 86.9%. This high rate indicates that **5h** strongly triggers apoptotic cell death via caspase‐3‐mediated mechanisms.

### 3.3 Molecular Docking

In in vitro enzyme studies conducted to elucidate the potential activity pathways of compounds showing efficacy against BC, it was determined that the compounds did not show significant activity on the aromatase enzyme, one of the main targets of BC; however, they exhibited inhibitory potential on the Akt1 enzyme. These findings suggested that the compounds may exhibit anticancer activity by triggering apoptosis via the PI3K/Akt signaling pathway. Accordingly, molecular docking studies were performed using PI3K (PDB ID: 4JPS) (Furet et al. 2013) and Akt1 (PDB ID: 3O96) (Wu et al. 2010) targets. Table 4 shows the molecular docking scores obtained for both crystal structures and the amino acid residues involved in ligand‐protein interactions. The binding positions of compounds **5a–5g** at the active sites of Akt‐1 (PDB ID: 3O96) and PI3K (PDB ID: 4JPS) enzymes are presented in Figures S33–S36, respectively. As a result of redocking analyses performed to validate the docking protocol, the overlapping binding position of compound **5h** at the PI3K (PDB ID: 4JPS) active site with the experimental ligand is shown in Figure S37, and the overlapping binding position at the Akt‐1 (PDB ID: 3O96) active site with the experimental ligand is shown in Figure S38. Furthermore, graphs showing the correlation between the docking scores of the compounds and the MM/GBSA binding free energies are presented in Figures S39 and S40.

**TABLE 4:** Docking scores (kcal/mol) and key binding residues of compounds **5a–5h** against target proteins (3O96 and 4JPS).

Comp.	PDB ID:3O96			PDB ID:4JPS		
	Docking scores	MM/GBSA	Key binding residues	Docking Scores	MM/GBSA	Key binding residues
**5a**	−7.420	−56.56	Trp80 (pi‐pi interaction) Lys268 (cation‐pi interaction)	−6.567	−54.99	—
**5b**	−8.038	−47.52	Asn54 (hydrogen bond) Trp80 (pi‐pi interaction) Lys268 (halogen bond) Tyr326 (hydrogen bond)	−5.968	−58.07	Tyr836 (pi‐pi interaction)
**5c**	−7.713	−52.59	Ser56 (hydrogen bond) Ala58 (hydrogen bond) Trp80 (pi‐pi interaction) Lys268 (cation‐pi interaction)	−6.432	−50.43	Tyr780 (pi‐pi interaction) His855 (pi‐pi interaction) Asn853 (halogen bond) Lys802 (hydrogen bond)
**5d**	−7.301	−50.75	Asn54 (hydrogen bond) Trp80 (pi‐pi interaction) Ile290 (halogen bond)	−6.843	−52.88	Tyr836 (pi‐pi interaction) Ser854 (hydrogen bond)
**5e**	−7.789	−45.52	Arg273 (cation‐pi interaction)	−6.583	−47.15	Tyr836 (pi‐pi interaction)
**5f**	−8.474	−38.55	Asn54 (hydrogen bond) Trp80 (pi‐pi interaction) Lys268 (halogen bond) Tyr326 (hydrogen bond)	−5.654	−47.66	Ser854 (hydrogen bond)
**5g**	−8.756	−52.44	Asn54 (hydrogen bond) Trp80 (pi‐pi interaction) Thr211 (halogen bond) Ile290 (halogen bond) Tyr326 (hydrogen bond)	−5.949	−41.36	Tyr836 (pi‐pi interaction)
**5h**	−8.609	−46.48	Asn54 (hydrogen bond) Trp80 (pi‐pi interaction) Leu213 (halogen bond) Tyr326 (hydrogen bond)	−7.161	−57.98	Asp810 (halogen bond) Tyr836 (pi‐pi interaction)

**FIGURE 3:**
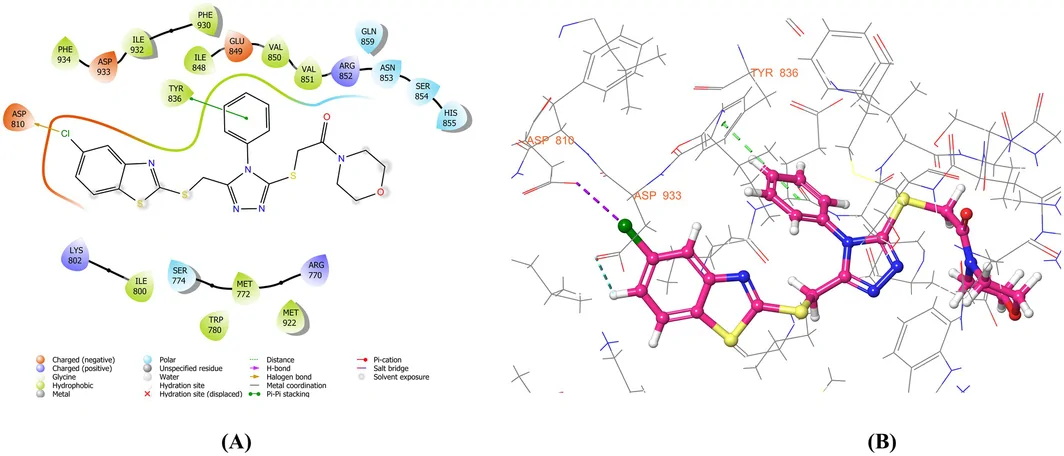
Two‐dimensional (2D) and three‐dimensional (3D) representation of the interactions of compound **5h** with the active site of the PI3K (PDB ID: 4JPS).

First, the binding positions obtained using PDB ID: 4JPS (Furet et al. 2013) for the PI3K target are presented in Figure 3. Examination of the obtained positions revealed the formation of a halogen bond between the chlorine atom in the benzothiazole ring of compound **5h** and the hydroxyl group of the Asp810 residue. Furthermore, a π–π interaction occurred between the phenyl group attached to the triazole ring of compound **5h** and the phenyl ring of residue Tyr836. In addition, it was determined that an aromatic hydrogen bond formed between the benzothiazole ring of compound **5h** and the carbonyl group of residue Asp933.

**FIGURE 4:**
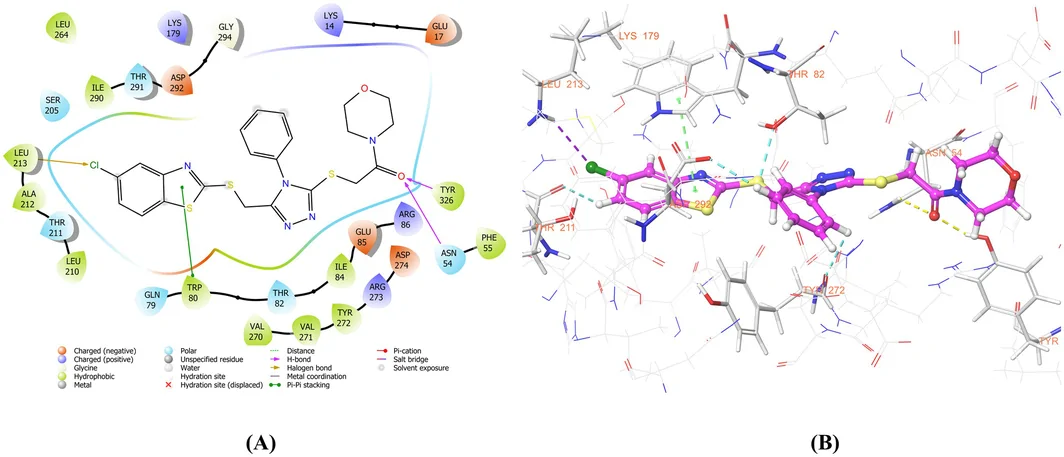
Two‐dimensional (2D) and three‐dimensional (3D) representation of the interactions of compound **5h** with the active site of the AKT‐1 (PDB ID: 3O96).

Secondly, molecular docking studies were performed using PDB ID: 3O96 (Wu et al. 2010) for the AKT‐1 target (Figure 4). Analysis of the results revealed the formation of a halogen bond between the chlorine atom in the benzothiazole ring of the compound coded **5h** and the amino group of the Leu213 amino acid. Furthermore, a π–π interaction was observed between the benzothiazole ring and the indole ring of the Trp80 residue. It was determined that the carbonyl group of the compound forms a hydrogen bond with the hydroxyl group of the Tyr326 residue and a second hydrogen bond with the amino group of the Asn54 amino acid.

In addition to these interactions, an aromatic hydrogen bond was observed between the benzothiazole ring and the carbonyl group of the Thr211 residue; and two separate aromatic hydrogen bonds were found between the mercapto bridge and the hydroxyl groups of the Asp292 and Thr82 residues. Furthermore, it was determined that an aromatic hydrogen bond is formed between the phenyl group attached to the triazole ring and the carbonyl group of the Tyr272 residue. Thus, compound **5h** was found to exhibit a strong binding profile in the Akt1 active site, supported by two hydrogen bonds, one halogen bond, one π–π interaction, and four aromatic hydrogen bonds.

### 3.4 Molecular Dynamics Simulation

Molecular dynamics studies were performed on both the **5h+4JPS** and **5h+3O96** complexes. The complexes obtained from docking studies were prepared using the POPE membrane model at a temperature of 310.55 K and a selection area of 20 Å, followed by molecular dynamics simulations for 100 ns.

**FIGURE 5:**
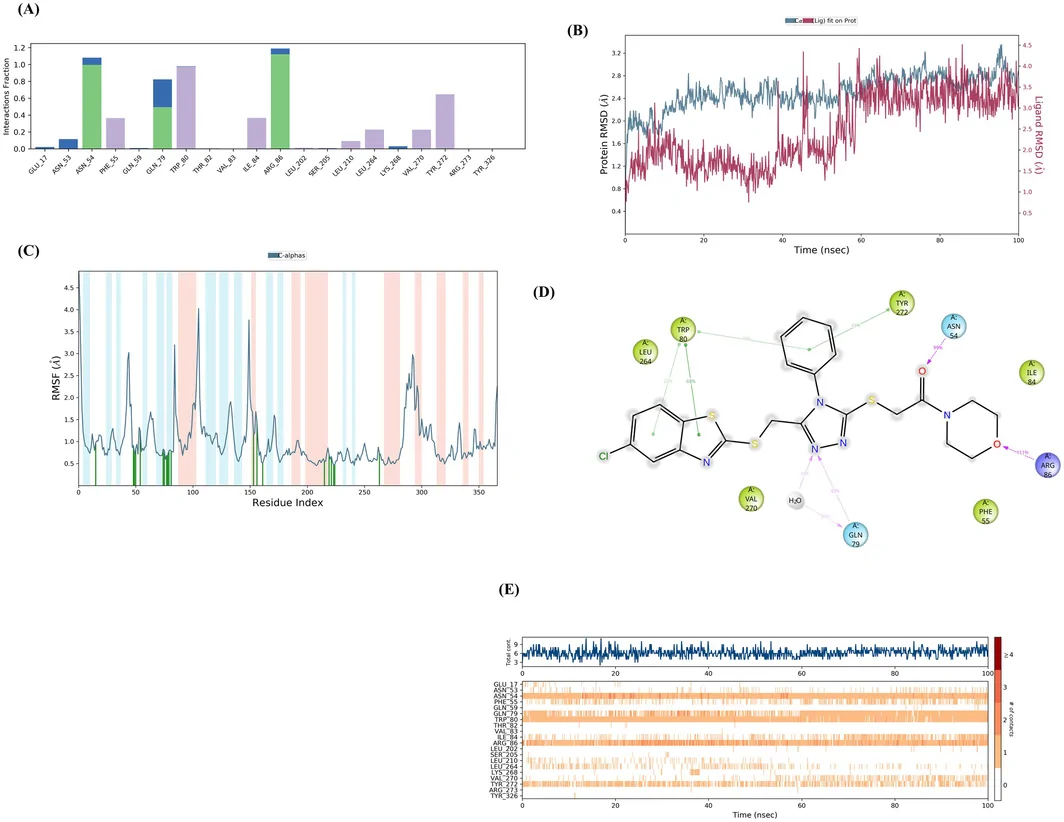
Molecular dynamic results of complex **5h+4JPS** (A) Interaction fractions by residue during the simulation (Blue: Water mediated H‐bond; green: H‐bond, purple: Hydrophobic interaction) (B) RMSD profiles of the MD simulations (100 ns) (C) RMSF profiles of the MD simulations (100 ns) (D) 2D diagram of the interaction strengths (cut off 10%) (100 ns) (E) Amino acid interactions timeline graphic of complex.

First, the results obtained for the **5h+4JPS** complex are presented in Figure 5. The RMSD value of the complex remained at approximately 2.4 Å throughout the simulation (Figure 5B), indicating that the ligand‐enzyme complex maintained its stability throughout the dynamic process. RMSF analysis (Figure 5C) revealed that most amino acids constituting the active site exhibited low fluctuation values. The low RMSF values of critical residues, particularly those located in the ATP binding region such as Lys802 (0.73 Å), Tyr836 (0.60 Å), Val851 (0.72 Å), Gln859 (1.18 Å), Met922 (0.73 Å), Ile932 (0.75 Å), and Asp933 (0.73 Å), indicate that the complex is stably maintained in the active site. A two‐dimensional representation of the interactions is given in Figure 5D.

When the time‐dependent analysis of protein‐ligand contacts was examined, it was determined that compound **5h** interacts continuously with the Trp780 amino acid located in the PI3Kα active site for 100 ns (Figure 5E). Similarly, the interaction with Arg770 was continuously maintained after approximately 60 ns of the simulation. In addition, intermittent but persistent contacts were observed with the amino acids Thr856, Gln859, and Ile932 throughout the simulation period. In particular, the conserved double aromatic hydrogen bond interactions with Val851 in the hinge region, which plays a crucial role in the binding of PI3Kα inhibitors, demonstrate the strong and stable incorporation of the ligand into the ATP binding pocket. Furthermore, the sustained hydrogen bond observed with Gln859 significantly contributed to the stabilization of the complex and helped the ligand maintain its binding conformation in the active site. These persistent interactions with Val851 and Gln859 provide significant molecular evidence supporting the potential of compound **5h** as a PI3Kα inhibitor.

**FIGURE 6:**
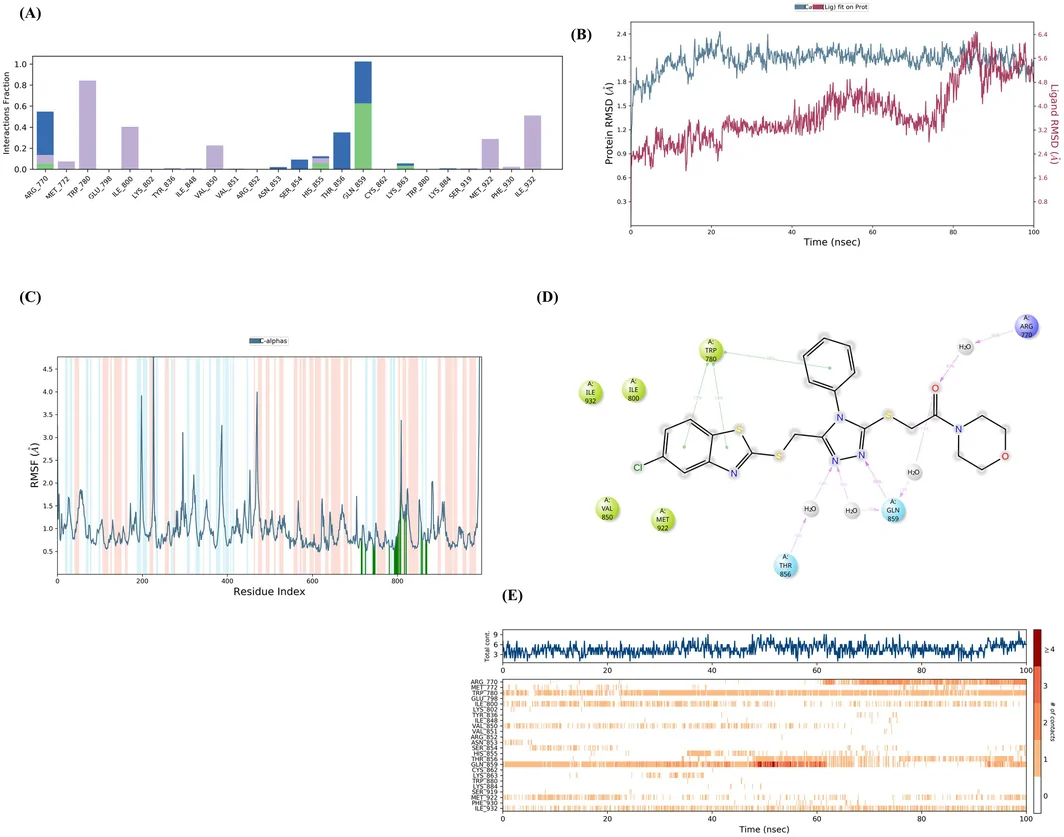
Molecular dynamic results of complex **5h+3O96**. (A) Interaction fractions by residue during the simulation (Blue: Water mediated H‐bond; green: H‐bond, purple: Hydrophobic interaction) (B) RMSD profiles of the MD simulations (100 ns) (C) RMSF profiles of the MD simulations (100 ns) (D) 2D diagram of the interaction strengths (cut off 10%) (100 ns) (E) Amino acid interactions timeline graphics of complexes.

Secondly, dynamic studies were performed on the **5h+3O96** complex for 100 ns. The results obtained are presented in Figure 6. The RMSD value of the complex remained at approximately 3.2 Å throughout the simulation (Figure 6B), indicating that the ligand‐enzyme complex maintained its stability throughout the dynamic process. In the RMSF analysis (Figure 6C), it was determined that most amino acids forming the active site exhibited low fluctuation values. These fluctuation values can be listed as follows: Glu17 (0.97 Å), Asn53 (0.91 Å), Asn54 (0.71 Å), Phe55 (0.84 Å), Gln79 (0.83 Å), Trp80 (0.80 Å), Thr82 (0.68 Å), Val83 (0.68 Å), Ile84 (0.79 Å), Arg86 (0.78 Å), Leu202 (1.16 Å), Ser205 (1.44 Å), Leu210 (0.51 Å), Leu264 (0.55 Å), Lys268 (0.71 Å), Val270 (0.66 Å), Tyr272 (0.46 Å), Arg273 (0.47 Å), and Tyr326 (0.74 Å). A two‐dimensional representation of the interactions is given in Figure 6D. When the time‐dependent analysis of protein‐ligand contacts was examined, it was determined that compound **5h** interacts continuously for 100 ns with the amino acids Asn54, Gln79, Trp80, Arg86, and Tyr272 located in the Akt1 active site (Figure 6E). A 100 ns molecular dynamics simulation of the **5h+3O96** complex revealed that compound **5h** exhibits a stable binding profile at the Akt1 active site. The maintenance of uninterrupted contacts with the amino acids Asn54, Gln79, Trp80, Arg86, and Tyr272 throughout the simulation indicates that the ligand maintains its stable position in the binding pocket. In particular, the ongoing interactions with Trp80 and Tyr272 suggest that the aromatic skeleton of the compound is stabilized at the active site by hydrophobic and π‐based contacts. Furthermore, the aromatic hydrogen bond maintained with Asp292 for 100 ns significantly contributed to complex stability as an important interaction associated with the Akt1 catalytic site. The subsequent cleavage of the aromatic hydrogen bond observed with Thr211 in the first 50 ns of the simulation suggests that the ligand exhibits limited conformational rearrangement over time. However, sustained contacts with other critical amino acids and the ongoing aromatic hydrogen bond with Asp292 support the stability of compound **5h** in the Akt1 active site and its Akt1 inhibition potential.

## 4 Conclusions

In this study, novel benzothiazole‐triazole derivatives were successfully synthesized, and their structures were characterized using NMR and HRMS analyses. Evaluation of the biological activities of the synthesized compounds revealed that all exhibited varying levels of cytotoxic activity against BC cells. As high cytotoxic activity and low toxicity towards healthy cells are important criteria in anticancer agent development, selectivity was evaluated using NIH/3T3 cells, and selectivity index values were calculated.

The biological results showed that compound **5h** stood out from the other derivatives due to its strong anticancer activity and greater selectivity towards healthy cells. Enzyme inhibition studies indicated that, while this compound did not have a significant effect on aromatase, it exhibited strong inhibitory properties against the Akt1 enzyme.

Further mechanistic studies revealed that compound **5h** strongly induced apoptotic cell death in MCF‐7 cells. Annexin V/PI analyses showed a significant increase in the apoptotic cell population, while the low necrosis rate supported the conclusion that the cytotoxic effect occurred through programmed cell death. The loss of mitochondrial membrane potential and high levels of caspase‐3 activation indicated that the apoptotic process proceeded via the mitochondria‐mediated intrinsic pathway.

In conclusion, among the synthesized benzothiazole‐triazole derivatives, the **5h** compound stands out for its high anticancer activity, advantageous selectivity profile, strong Akt1 inhibition, and mechanistic properties that trigger apoptosis. The findings indicate that the **5h** compound could be a promising precursor molecule for the development of novel Akt1‐targeted therapeutic agents for BC treatment. However, further in vivo studies and detailed pharmacokinetic evaluations are needed to more comprehensively elucidate the pharmacological potential of the compound.

The enzyme inhibition, molecular modeling, and cellular analysis results obtained in this study support the idea that the biological effects of compound **5h** may be related to AKT inhibition. However, to validate the proposed mechanism at the cellular level, evaluation of signaling pathway proteins such as p‐AKT, PI3K, mTOR, and GSK3β using Western blot analysis is planned for future research.

## Author Contributions

**Bilge Çiftçi:** writing – original draft, formal analysis, methodology, data curation. **Zeynep Gülenç:** writing – original draft, methodology, formal analysis, data curation. **Büşra Korkut Çelikateş:** investigation, writing – original draft, formal analysis, writing – review and editing. **Derya Osmaniye:** conceptualization, investigation, writing – review and editing, software, supervision, writing – original draft.

## Funding

Anadolu University Project coded BGT‐2023‐2345 was used as a funding source for the supply of materials used in the cytotoxicity part of the study.

## Conflicts of Interest

The authors declare no conflicts of interest.

## Supporting information


**Figure S1:** ^1^H‐NMR spectra of compound **5a**.
**Figure S2:** ^13^C‐NMR spectra of compound **5a**.
**Figure S3:** HRMS spectra of compound **5a**.
**Figure S4:** ^1^H‐NMR spectra of compound **5b**.
**Figure S5:** ^13^C‐NMR spectra of compound **5b**.
**Figure S6:** HRMS spectra of compound **5b**.
**Figure S7:** ^1^H‐NMR spectra of compound **5c**.
**Figure S8:** ^13^C‐NMR spectra of compound **5c**.
**Figure S9:** HRMS spectra of compound **5c**.
**Figure S10:** ^1^H‐NMR spectra of compound **5d**.
**Figure S11:** ^13^C‐NMR spectra of compound **5d**.
**Figure S12:** HRMS spectra of compound **5d**.
**Figure S13:** ^1^H‐NMR spectra of compound **5e**.
**Figure S14:** ^13^C‐NMR spectra of compound **5e**.
**Figure S15:** HRMS spectra of compound **5e**.
**Figure S16:** ^1^H‐NMR spectra of compound **5f**.
**Figure S17:** ^13^C‐NMR spectra of compound **5f**.
**Figure S18:** HRMS spectra of compound **5f**.
**Figure S19:** ^1^H‐NMR spectra of compound **5g**.
**Figure S20:** ^13^C‐NMR spectra of compound **5g**.
**Figure S21:** HRMS spectra of compound **5g**.
**Figure S22:** ^1^H‐NMR spectra of compound **5h**.
**Figure S23:** ^13^C‐NMR spectra of compound **5h**.
**Figure S24:** HRMS spectra of compound **5h**.
**Figure S25:** Purity report of compound **5a**.
**Figure S26:** Purity report of compound **5b**.
**Figure S27:** Purity report of compound **5c**.
**Figure S28:** Purity report of compound **5d**.
**Figure S29:** Purity report of compound **5e**.
**Figure S30:** Purity report of compound **5f**.
**Figure S31:** Purity report of compound **5g**.
**Figure S32:** Purity report of compound **5h**.
**Figure S33:** Docking poses of compounds **5a–5d** with Akt‐1 enzyme active site (PDB ID:3O96).
**Figure S34:** Docking poses of compounds **5e–5h** with Akt‐1 enzyme active site (PDB ID:3O96).
**Figure S35:** Docking poses of compounds **5a–5d** with PI3K enzyme active site (PDB ID:4JPS).
**Figure S36:** Docking poses of compounds **5e–5h** with PI3K enzyme active site (PDB ID:4JPS).
**Figure S37:** Redocking poses of compound **5h** with PI3K enzyme active site (PDB ID:4JPS).
**Figure S38:** Redocking poses of compound **5h** with AKT enzyme active site (PDB ID:3O96).
**Figure S39:** PDB ID: 4JPS Correlation between molecular docking scores for protein‐ligand complexes and MM/GBSA binding free energies. Each point represents a test compound.
**Figure S40:** PDB ID: 3O96 Correlation between molecular docking scores for protein‐ligand complexes and MM/GBSA binding free energies. Each point represents a test compound.

## Data Availability

The data that supports the findings of this study are available in Figures S1–S40 of this article.
