# Supplementary material for: Synthesis, Structural Characterization, and Investigation of Biological Effects of Novel Benzothiazole‐Triazole Derivatives Designed as AKT Kinase Inhibitors
Source: Chem Biol Drug Des. 2026 Sep 17;108(3):e70404. doi: 10.1111/cbdd.70404 (PMC13586420; doi:10.1111/cbdd.70404)
Supplement: Supplementary file 1 [file CBDD-108-e70404-s001.docx]

**SUPPORTING INFORMATION**

Synthesis, Structural Characterization, and Investigation of Biological Effects of Novel Benzothiazole-Triazole Derivatives Designed as AKT Kinase Inhibitors

Bilge Çiftçi^1,2^, Zeynep Gülenç^1^, Büşra Korkut Çelikateş^3^, Derya Osmaniye^4,5*^

*^1^Institute of Graduate Education, Anadolu University, Eskişehir 26470, Turkey*

*^2^Department of Medical Services Techniques Services, Vocational School of Health Services, Bilecik Seyh Edebali University, Bilecik 11230, Turkey*

*^3^Department of Pharmaceutical Toxicology, Faculty of Pharmacy, Anadolu University, Eskişehir 26470, Turkey*

*^4^Department of Pharmaceutical Chemistry, Faculty of Pharmacy, Anadolu University, Eskişehir 26470, Turkey*

*^5^Central Research Laboratory, Faculty of Pharmacy, Anadolu University, Eskişehir 26470, Turkey*

* Correspondence: dosmaniye@anadolu.edu.tr

*Tel*: +90- *Fax*: +90-222 335 05 80-3778

*Address*: Anadolu University, Faculty of Pharmacy, Department of Pharmaceutical Chemistry, 26470, Eskişehir, Turkey.

**Figure S1.** ^1^H-NMR spectra of compound **5a**

**Figure S2.** ^13^C-NMR spectra of compound **5a**

**Figure S3.** HRMS spectra of compound **5a**

**Figure S4.** ^1^H-NMR spectra of compound **5b**

**Figure S5.** ^13^C-NMR spectra of compound **5b**

**Figure S6.** HRMS spectra of compound **5b**

**Figure S7.** ^1^H-NMR spectra of compound **5c**

**Figure S8.** ^13^C-NMR spectra of compound **5c**

 **Figure S9.** HRMS spectra of compound **5c**

**Figure S10.** ^1^H-NMR spectra of compound **5d**

**Figure S11.** ^13^C-NMR spectra of compound **5d**

 **Figure S12.** HRMS spectra of compound **5d**

**Figure S13.** ^1^H-NMR spectra of compound **5e**

**Figure S14.** ^13^C-NMR spectra of compound **5e**

 **Figure S15.** HRMS spectra of compound **5e**

**Figure S16.** ^1^H-NMR spectra of compound **5f**

**Figure S17.** ^13^C-NMR spectra of compound **5f**

 **Figure S18.** HRMS spectra of compound **5f**

**Figure S19.** ^1^H-NMR spectra of compound **5g**

**Figure S20.** ^13^C-NMR spectra of compound **5g**

 **Figure S21.** HRMS spectra of compound **5g**

**Figure S22.** ^1^H-NMR spectra of compound **5h**

**Figure S23.** ^13^C-NMR spectra of compound **5h**

 **Figure S24.** HRMS spectra of compound **5h**

**Figure S25.** Purity report of compound **5a**

**Figure S26.** Purity report of compound **5b**

**Figure S27.** Purity report of compound **5c**

**Figure S28.** Purity report of compound **5d**

**Figure S29.** Purity report of compound **5e**

**Figure S30.** Purity report of compound **5f**

**Figure S31.** Purity report of compound **5g**

**Figure S32.** Purity report of compound **5h**

**Figure S33.** Docking poses of compounds **5a-5d** with Akt-1 enzyme active site (PDB ID:3O96)

**Figure S34.** Docking poses of compounds **5e-5h** with Akt-1 enzyme active site (PDB ID:3O96)

**Figure S35.** Docking poses of compounds **5a-5d** with PI3K enzyme active site (PDB ID:4JPS)

**Figure S36.** Docking poses of compounds **5e-5h** with PI3K enzyme active site (PDB ID:4JPS)

**Figure S37.** Redocking poses of compound **5h** with PI3K enzyme active site (PDB ID:4JPS)

**Figure S38.** Redocking poses of compound **5h** with AKT enzyme active site (PDB ID:3O96)

**Figure S39.** PDB ID: 4JPS Correlation between molecular docking scores for protein-ligand complexes and MM/GBSA binding free energies. Each point represents a test compound.

**Figure S40.** PDB ID: 3O96 Correlation between molecular docking scores for protein-ligand complexes and MM/GBSA binding free energies. Each point represents a test compound.
